# Effects of pulmonary rehabilitation therapy combined with conventional drugs on BODE and pulmonary function indexes in elderly patients with interstitial pneumonia

**DOI:** 10.12669/pjms.38.6.5748

**Published:** 2022

**Authors:** Zhen Zhang, Yumei Tian, Jing Yang

**Affiliations:** 1 Zhen Zhang Department of Respiratory and Critical Care Medicine, Wuhan Fourth Hospital, Wuhan 430000, Hubei Province, P.R. China; 2 Yumei Tian School of Nursing, Hunan University of Medicine, Huaihua 418000, Hunan Province, P.R. China; 3 Jing Yang Department of Pediatrics, Wuhan Fourth Hospital, Wuhan Fourth Hospital, Wuhan 430000, Hubei Province, P.R. China

**Keywords:** Pulmonary rehabilitation, Conventional drugs, Interstitial pneumonia, Curative effect, Pulmonary function

## Abstract

**Objectives:**

To investigate the effect of pulmonary rehabilitation therapy combined with conventional drugs on BODE and pulmonary function (PFT) indexes in elderly patients with interstitial pneumonia.

**Methods:**

Records of 89 elderly patients with interstitial pneumonia treated in the First Affiliated Hospital of Hunan Medical College from April 2020 to April 2021 were retrospectively selected. Of them, 41 patients received conventional drug treatment (Group-I) and 48 patients received pulmonary rehabilitation treatment and conventional drug treatment (Group-II). The clinical efficacy of therapy, BODE and PFT indexes of the two groups were compared.

**Results:**

The total efficacy of patients in the Group-II (93.75%) was significantly higher than that of Group-I (75.61%) (P<0.05). After the treatment, the BODE index score of Group-II was lower than that of Group-I, and the forced vital capacity (FVC), forced expiratory volume in the first second (FEV1) and peak expiratory flow rate (PEF) were higher than those of Group-I (P<0.001).

**Conclusion:**

Combination of pulmonary rehabilitation therapy and conventional drugs can further improve the curative effect of the treatment and help to improve BODE and PFT indexes in patients with senile interstitial pneumonia.

## INTRODUCTION

Interstitial pneumonia is a variation of diffuse pulmonary disease, which mostly occurs in the elderly. It can involve the alveolar wall, alveolar septum and surrounding blood vessels, and is caused by a variety of diseases without specific symptoms. Further progress of the disease can lead to progressive reduction of PFT, decline in exercise endurance and even respiratory failure.^1^,^2^ At present, the pathogenesis of interstitial pneumonia has not been fully clarified clinically, but studies suggest that it is related to the damage to the alveolar wall and lung stroma, caused by pathogenic factors, and accompanied by the inflammatory reaction. Inflammatory cells such as neutrophils and macrophages, and inflammatory mediators can directly damage the lung tissue.^3^,^4^ Currently, there are no specific drugs for the treatment of interstitial pneumonia. Nidanib and pirfenidone are among the approved drugs for the treatment of the disease, and acetylcysteine and glucocorticoid are commonly used symptomatic drugs. However, while the administration of these drugs can alleviate symptoms and delay the progression of the disease, it cannot reverse pulmonary fibrosis.^5^,^6^ Non-drug therapies, such as pulmonary rehabilitation therapy and home oxygen therapy, are often used in the treatment of interstitial pneumonia. Pulmonary rehabilitation therapy can promote the improvement of PFT and cardiopulmonary endurance through comprehensive multidisciplinary knowledge, exercise, breathing and other training techniques that are based on all-inclusive evaluation of patients’ PFT and psychological support.^7^

The purpose of this study was to explore the efficacy of pulmonary rehabilitation therapy combined with conventional drugs in elderly patients with interstitial pneumonia, and to assess the improvement of PFT in order to provide further reference for clinicians.

## METHODS

Medical records of 89 elderly patients (59 males and 30 females) with interstitial pneumonia, treated in our hospital from April 2020 to April 2021, were retrospectively collected.

### Inclusion criteria:

• Meeting diagnostic criteria of interstitial pneumonia;^8^
• Age>60 years;^9^
• No other cardiopulmonary diseases;
• Complete medical records.

###  Exclusion criteria:

• Patients with cardiopulmonary insufficiency;
• Patients with other serious basic diseases, liver and kidney dysfunction and malignant tumors;
• Recent peptic ulcer and active bleeding;

### Ethical Approval

This study was approved by the medical ethics Committee of the First Affiliated Hospital of Hunan Medical College (No.: 2020-022-02; Date: January 4, 2021).

According to the treatment records of patients, they were retrospectively divided into two treatment groups. Group-I included patients that received the routine drug treatment: prednisone (Zhejiang Xianju Pharmaceutical Co., Ltd., H33021207) 0.75mg/(kg·d), oral, once a day, reducing the dose of 5mg every month; Azathioprine (Heumann pcsgmb, H20100042) 50mg/ day, oral, increase the dosage by 25mg every two weeks until it reaches 150mg/day; Acetylcysteine (Hainan Zanbang Pharmaceutical Co., Ltd., H20000472) 600mg/time, oral, three times a day.

Group-II included patients that received pulmonary rehabilitation treatment in addition to the same treatment scheme as Group-I. Treatment regimen included 7g/kg protein intake per day, maintained low flow oxygen support, 6~8h/day, guided exercise and breathing training, including active joint activities and parallel breathing training.

### Breathing training techniques

Abdominal breathing (bend both knees to relax abdominal muscles, inhale to bulge the abdomen, hold your breath for 1~2S, exhale slowly, repeat three to four times, twice a day); Thoracic dilation breathing (inhale to promote thoracic dilation, hold your breath for 1~2 second, exhale slowly through your mouth, repeat three to four times, twice a day); Forced exhalation (inhale normally, open the glottis, exhale and make a “Ho” sound, repeat three to four times, twice a day). Psychological intervention: gradual introduction of the relevant information about the disease and the treatment methods, alleviation of the anxiety, tension, worry and other emotions caused by insufficient cognition of disease knowledge, targeted comfort, encouragement, patient guidance, and implementation of relaxation training intervention for patients (patients in the supine position are guided to relax, listen to light music and take a deep breath, relax, releasing bad emotions and changing them into positive emotions to promote active cooperation with relevant clinical interventions).

After three months of treatment, 89 patients were followed up by telephone or through outpatient services to observe the curative effect, calculate the BODE index and perform PFT test. Efficacy criteria were as follows:^10^ treatment was considered markedly effective if the patient’s clinical manifestations disappeared; the treatment was considered effective if after the treatment, the clinical manifestations of patients were alleviated; the treatment was considered ineffective if after the treatment, there was no remission of the symptoms or further aggravation. Total efficacy,was calculated as follows:(number of markedly effective cases+number of effective cases)/total number of people. BODE index included four variables:^11^,^12^ body mass index(B), degree of airflow obstruction(O), dyspnea index(D) and exercise ability(E). B>21kg/m^2^ is 0; B≤21kg/m^2^ is one. O>65% is zero point; 50%≤O≤64% is one point, 36%≤O≤49% is two points, O≤35% is three points. D≤1 time/minute is zero point; D=2 times/minute is one point; D=3 times/minute is two points; D=4 times/minute is three points. E≥350m equals zero points, 250≤E≤349m is one point; 150≤E≤249m is two points; E ≤149 is three points. PFT indexes were measured by the PFT test system (Masterscreen IOS, Yeager, Germany), including forced vital capacity (FVC), forced expiratory volume in the first second (FEV1) and peak expiratory flow rate (PEF).

The total efficacy, BODE and PFT indexes of the two groups were compared. The data were processed by Spss22.0, and the counting data were expressed by [n (%)], the test method was *χ^2^*; (*x̅*±s) was used to represent the measurement data; t-test was used for normal distribution and rank sum test-for non-normal distribution, P<0.05 was considered statistically significant.

## RESULTS

A total of 89 patients met the inclusion criteria. Of them, 41 patients were treated with the routine drug treatment (Group-I) and 48 patients treated with the routine drug treatment in combination with pulmonary rehabilitation therapy (Group-II). There was no significant difference in the basic clinical characteristics of patients in the two groups (P>0.05) (Table-I). The total efficacy,of Group-II (93.75%) was significantly higher than that of Group-I (75.61%) (P<0.05), as shown in Table-II. There was no difference in BODE index between the two groups before the treatment. After the treatment BODE indexes of both groups decreased (P<0.05), and the Group-II treatment scheme was associated with significantly lower BODE index than Group-I (P<0.05) (Table-III). There was no significant difference in the PFT indexes (FVC, FEV1 and PEF) between the two groups before the treatment (P>0.05), but they all increased after the treatment (P<0.05), and patients in Group-II had significantly higher PFT indexes than patients in Group-I (P<0.05) (Table-IV).

**Table-I:** Comparison of clinical characteristics between the two groups [n (%),(*x̅*±s ].

Treatment mode	n	Gender(male/Female)	Age(year)	Clinical manifestations [n (%)]			

				Cough	Expectoration	Difficulty breathing	Velcro rales
Group-I	41	24/17	65.61±3.08	35(85.36)	32(78.04)	30(73.17)	30(73.17)
Group-II	48	30/18	66.02±3.01	41(85.42)	39(81.25)	37(77.08)	32(66.67)
Χ^2^/t	-	0.146	0.635	0.000	0.140	0.182	0.443
P	-	0.703	0.527	0.995	0.708	0.670	0.506

**Table-II:** Comparison of the total effective rate of the two groups after treatment [n(%)].

Treatment mode	n	Clinical efficacy			Total

		Markedly effective	Efficient	Invalid	
Group-I	41	11(26.83)	20(48.78)	10(24.39)	31(75.61)
Group-II	48	25(52.8)	20(41.67)	3(6.25)	45(93.75)
Χ^2^	-	-	-	-	5.834
P	-	-	-	-	0.016

**Table-III:** Comparison of BODE index between the two groups before and after treatment ( (*x̅*, points).

Treatment mode(n)	B		O		D		E	

	Before treatment	After treatment	Before treatment	After treatment	Before treatment	After treatment	Before treatment	After treatment
Group-I(n=41)	1.28±0.35	1.11±0.29^a^	2.42±0.32	1.96±0.25^a^	2.42±0.30	1.94±0.23^a^	2.44±0.34	1.93±0.27^a^
Group-II(n=48)	1.29±0.38	0.93±0.25^ab^	2.45±0.34	1.18±0.26^ab^	2.44±0.31	1.26±0.24^ab^	2.45±0.37	1.19±0.29^ab^
t	0.128	3.196	0.433	13.937	0.376	13.255	0.202	12.142
P	0.899	0.002	0.666	<0.001	0.708	<0.001	0.841	<0.001

***Note:*** ^a^ compared with this group before treatment P<0.05, ^b^ compared with the control group P<0.05.

**Table-IV:** Comparison of PFT indexes of the two groups before and after the treatment ((*x̅*±s).

Treatment mode(n)	FVC(L)		FEV1(%)		PEF(L/S)	

	Before treatment	After treatment	Before treatment	After treatment	Before treatment	After treatment
Group-I (n=41)	2.21±0.39	2.46±0.34^a^	65.78±7.32	74.32±6.51^a^	2.89±0.26	3.25±0.22^a^
Group-II (n=48)	2.24±0.40	2.91±0.36^ab^	65.18±7.73	81.56±6.88^ab^	2.88±0.24	4.11±0.20^ab^
t	0.401	6.085	0.369	5.071	0.342	19.097
P	0.690	<0.001	0.712	<0.001	0.733	<0.001

***Note:*** ^a^ Compared with this group before treatment P<0.05, ^b^ compared with the control group P<0.05.

## DISCUSSION

In this study, we showed that pulmonary rehabilitation therapy in combination with conventional drugs led to significantly improved BODE and PFT indexes in patients with senile interstitial pneumonia, as compared to conventional therapy alone. Conventional drugs mainly include prednisone+azathioprine+acetylcysteine which can inhibit the inflammatory reaction, dissolve and promote the expectoration of sputum.^13^,^14^ Based on the comprehensive assessment of the patient’s condition, pulmonary rehabilitation therapy may additionally provide protein supplementation, oxygen inhalation, exercise and respiratory training guidance and psychological intervention. Protein supplementation can promote the improvement of immune function, while oxygen inhalation can improve the symptoms of dyspnea. Exercise and respiratory training guidance can further promote the improvement of exercise endurance and respiratory function.^15^ Dowman et al. found that pulmonary rehabilitation exercise training is effective patients with various types of interstitial lung disease. It can significantly improve patients’ dyspnea and overall condition and quality of life.^16^ Cerdan et al. also confirmed that pulmonary rehabilitation therapy can significantly improve the exercise ability and PFT indexes of patients with idiopathic pulmonary fibrosis, leading to improved satisfaction of patients.^17^ Cox NJ et al. studied the effect of rehabilitation plan on patients with asthma or COPD in a prospective clinical trial involving 87 patients. Among them, 43 patients only received routine drug treatment, and 44 patients received rehabilitation plan, which included the best medical treatment, combined with physical training, health education and psychological and social support. After three months, PFT indexes of patients that received a combine treatment were significantly improved, resulting in effectively improved prognosis.^18^ Our results are in agreement with these observations. We may speculate that when pulmonary rehabilitation therapy is combined with conventional drugs in the treatment of senile interstitial pneumonia, the two methods play a synergistic role, further promote the improvement of clinical efficacy, improve patients’ cough, expectoration, dyspnea and other related symptoms, promote the improvement of exercise endurance and PFT, and effectively improve patients’ prognosis.

Studies show that the average age of patients with interstitial pneumonia at the time of diagnosis is 67 years old. The relative reduction of muscle fibers and muscle atrophy in the elderly can reduce the strength of respiratory muscles. In addition, the reduction of elastic fibers in lung tissue and the increase of alveolar cavity can reduce the driving force of respiratory center and increase the risk of interstitial pneumonia.^19^ Moreover, compared with the young population, the immune function of elderly patients with interstitial pneumonia is lower. Under the influence of the massive release of alveolar macrophages and interstitial cell growth factor, there is abnormal activation and proliferation of fibroblasts that promote the progress of micro fibrosis, damage the alveoli and pulmonary vessels, leading to the serious decline in PFT indexes. At this stage, the clinical purpose of treating senile interstitial pneumonia is mainly anti-inflammatory and anti-fibrotic. Although it can effectively delay the progression of the disease, the effect of a single drug on the improvement of PFT is still poor.^20^ Our results suggest that rehabilitation training and home oxygen therapy can be effectively used in combination with conventional drug therapy for treating patients with senile human interstitial pneumonia, and can effectively promote the rehabilitation of patients’ PFT.

### Limitations of the study

The main limitation of this retrospective study is that the patients were followed up for only three months. Prospective and retrospective studies with larger sample size and longer follow-up are needed to further evaluate the efficiency of the two methods.

## CONCLUSION

A combination of pulmonary rehabilitation and conventional drug therapies significantly improve the curative effect of the treatment and further improve BODE and PFT indexes in patients with senile interstitial pneumonia. Our results can provide reference for optimizing care of patients with senile interstitial pneumonia.

### Authors’ contributions:

**ZZ:** Conceived and designed the study.

**YT & JY:** Collected the data and performed the analysis.

**ZZ:** Was involved in the writing of the manuscript and is responsible for the integrity of the study.

All authors have read and approved the final manuscript.
